# The colony forming efficiency assay for toxicity testing of nanomaterials—Modifications for higher-throughput

**DOI:** 10.3389/ftox.2022.983316

**Published:** 2022-09-07

**Authors:** Elise Rundén-Pran, Espen Mariussen, Naouale El Yamani, Elisabeth Elje, Eleonora Marta Longhin, Maria Dusinska

**Affiliations:** ^1^ Health Effects Laboratory, Department of Environmental Chemistry, NILU—Norwegian Institute for Air Research, Kjeller, Norway; ^2^ Norwegian Institute of Public Health, Department for Environmental Chemistry, Department of Air Quality and Noise, Oslo, Norway; ^3^ University of Oslo, Faculty of Medicine, Institute of Basic Medical Sciences, Department of Molecular Medicine, Oslo, Norway

**Keywords:** nanomaterials, cytotoxicity, cell viability, colony formation, CFE, 12-well format, hazard and risk assessment

## Abstract

To cope with the high number of nanomaterials manufactured, it is essential to develop high-throughput methods for *in vitro* toxicity screening. At the same time, the issue with interference of the nanomaterial (NM) with the read-out or the reagent of the assay needs to be addressed to avoid biased results. Thus, validated label-free methods are urgently needed for hazard identification of NMs to avoid unintended adverse effects on human health. The colony forming efficiency (CFE) assay is a label- and interference-free method for quantification of cytotoxicity by cell survival and colony forming efficiency by CFE formation. The CFE has shown to be compatible with toxicity testing of NMs. Here we present an optimized protocol for a higher-throughput set up.

## Introduction

The nanosize that gives rise to the highly advantageous properties of nanomaterials (NMs) designed for various products and purposes could also induce unintended effects on human health. To develop safe NMs, the safer-by-design (SbD) principle should be followed, whereby toxicity testing is performed in parallel with the development of the NMs (Yan et al., 2019; Sánchez Jiménez et al., 2022). Validated test methods for NM hazard identification are urgently needed, as standard toxicity test protocols often need modifications to avoid biased results. An important aspect of this is the potential interference of the NM with the read-out or reagents of the assay applied, due to the high reactivity of the NMs (MacCormack et al., 2021). This can be a challenge in optical detection methods (light absorption, fluorescence), metabolic assays (chemical reaction between the NMs and the assay components) and enzymatic assays (adsorption of assay molecules (e.g. antibodies, enzymes) on the particle surface) (Kroll et al., 2012; Guadagnini et al., 2013; Lee et al., 2022). Thus, label-free *in vitro* test methods are very beneficial to significantly reduce the likelihood of interaction and biased hazard identification of NMs. Due to the vast number of NM-based products, it is not possible to test all of them by standard assays, thus higher-throughput toxicity tests are needed.

The cytotoxic effects of chemicals, including NMs, can be determined by different endpoints, such as membrane integrity (e.g. trypan blue assay), metabolic activity (e.g. AlamarBlue, MTT, WST-1 assay), relative cell proliferation (e.g. relative cell growth assay) or label-free impedance analysis (e.g. xCELLigence system). Cell viability can also be measured by the ability of cells to survive and form colonies, which is the endpoint of the colony forming efficiency assay (CFE). Being non-colorimetric and non-fluorescent, the CFE assay is especially suitable for assessment of toxicity of NMs to avoid potential interference.

The CFE assay is applicable for most adherent mammalian cells in culture, and stable cell lines are mostly used. Individual cells are exposed, and each surviving cell will divide and form a colony. This allows for the quantification of cell survival/cell death, and also for the detection of cytostatic effects by evaluating the size of the colonies. Reduced colony size will reflect slowed cell proliferation and growth. The test method has similarities with the plating efficiency assay (part of the OECD test guideline 476), however, for the plating efficiency assay exposure is performed on a confluent cell population grown in monolayer.

The CFE assay was optimized and standardized some years ago for NMs testing by the JRC’s Nanobiosciences Unit and validated in a interlaboratory comparison study (Ponti et al., 2014), and it has been used with different *in vitro* systems to assess the cytotoxicity of a wide range of NMs e.g., gold NMs (Coradeghini et al., 2013), silver NMs (Locatelli et al., 2012; El Yamani et al., 2017), titanium oxide NMs (de Angelis et al., 2012; Fenoglio et al., 2013; El Yamani et al., 2017; Lee et al., 2022), zinc oxide NMs (de Angelis et al., 2012; El Yamani et al., 2017), silica NMs (Uboldi et al., 2012; Lee et al., 2022), multi-walled carbon nanotubes (Ponti et al., 2010), copper oxide (Lee et al., 2022), graphene (Won et al., 2022), nickel (Latvala et al., 2016), and cerium oxide NMs (El Yamani et al., 2017; Lee et al., 2022).

In this paper we provide the protocol for an optimized and miniaturized version of the CFE assay for higher through put, moving from Petri dishes to 6-well plates and further to 12-well plates. The assay is easy to perform, and time- and cost-efficient, and found to be very suitable for cytotoxicity testing of NMs. As in general for NM testing, specific considerations should be followed. Toxicity of NMs is dependent on physico-chemial properties, such as size, shape and surface coating. Thus, NMs need to be fully characterized, dispersible in culture medium and stability of the NM dispersion needs to be checked and reported (El Yamani et al., 2017; Elespuru et al., 2022).

## Materials and equipment

### Materials

Cells (adherent cell line), flasks 25 cm^2^ or/and 75 cm^2^, 12-well (or 6-well) plates, sterile plastic centrifuge tubes, microcentrifuge tubes, serological pipettes, pipettes and tips, cell culture medium (according to cell line) and additives (e.g serum, Penicillin-Streptomycin), trypsin-EDTA, methylene blue (CAS number 122965-43-9), filtration paper, phosphate buffered saline (PBS), CO_2_, distilled water, ethanol, Bürker chamber + Cover slips 22 × 22 mm/Cell counter slides, trypan blue stain 0.4%, ink pen or e-count pen.

### Equipment needed

Laminar flow hood, light microscope, automated cell counter*/*Bürker chamber, pipettes, CO_2_ incubator, refrigerator, water bath, vortex, autoclave.

### Solutions

**Preparation of methylene blue (1%)**: l g of methylene blue is dissolved in 100 ml of MilliQ water. Filter through filtration paper. It is not necessary to sterilize it. The solution can be kept at room temperature.

## Methods

**FIGURE 1:**
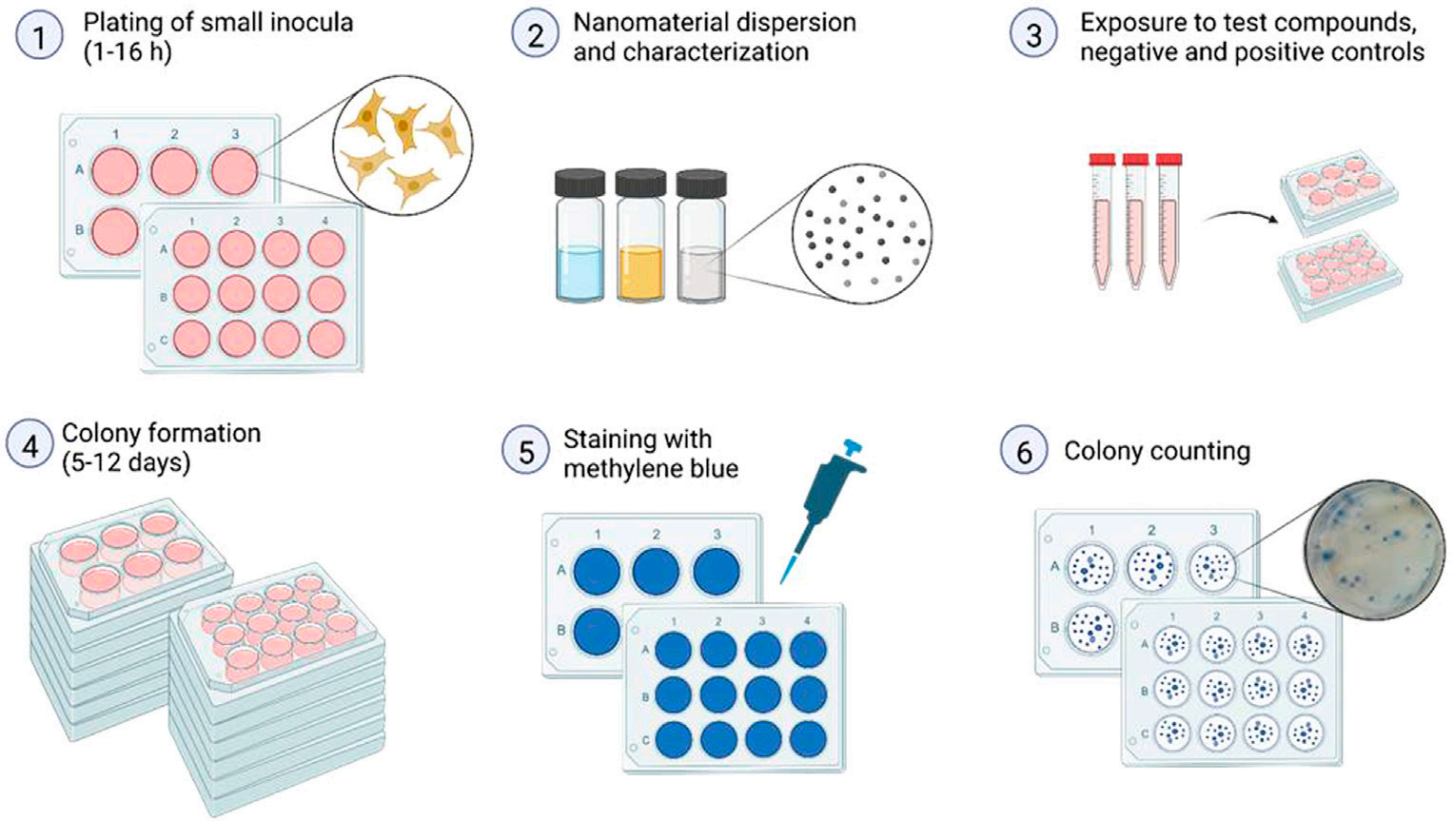
Graphical design of the colony forming efficiency (CFE) assay (Created with BioRender.com). 1. Trypsinize and count the cells. Seed the cells in correct density. It is important to mix the suspension prior to plating to ensure an even suspension of cells, as well as to spread the cells evenly in the wells. Remember to label both lid and the plate properly to avoid mix-up. Keep the cells in the incubator. 2. Prepare dispersion of NMs. Perform proper particle characterization. 3. Dilute NMs and controls in culture medium and add to the plates. Remember to make 2 × concentration since there is already half of medium in the well. 4. Leave the plates with the cells in the incubator to form colonies, normally 5–12 days 5. When colonies visible by eye are formed in negative control plates, the colonies should be stained in 1% methylene blue. Add 20 µl of methylene blue into each well and leave for minimum 30 min. Remove the staining solution into waste bottle. To reduce background staining, the plates can be rinsed carefully with water after staining. Leave the plates to dry. 6. Count the colonies. h, hours; d, days.

The CFE assay is performed on individual mammalian cells plated out in small inoculum (i.e. 25-200 cells per well) on 12- (or 6) well plates at 1–16 h (h), depending upon growth rate of the cells, before treatment. The cells should not divide after seeding before exposure. Then, cells are exposed to the test compound, positive and negative controls and cultured to allow for colony formation, generally for 5–12 days (d), depending on cell type and their doubling time. The colonies are stained and counted manually or by automated scoring. A brief outline of the steps is given in Figure 1, followed by subsections with more detailed description.

### Cell lines and preparation of culture

Human or mammalian cells growing attached to the surface with high cloning efficiency, such as V79, A549 or HepG2 cells (El Yamani et al., 2017; Lee et al., 2022), are commonly used with the assay. Any adherent cells growing with high cloning efficiency can be used. Cells are cultivated in complete culture medium and incubated in culture dishes or flasks in a cell incubator with humidified atmosphere at 37°C, 5% of CO_2_ as described in the standard operating procedure (SOP) for cultivation of the cell line.

Cells are thawed, put into culture medium and cultivated in a cell incubator.

### Seeding of cells for exposure

The cells should be sub-cultured at least 2–3 times before being seeded for exposure. Cells should be taken in the exponential growth phase (50–80% confluency) in low passage (max P15). Briefly, seed cells in 12-well plates in low inoculum at 1–16 h before exposure. The time is selected depending on the generation time for the cells, as the cells should not divide between seeding and exposure to be able to expose individual cells.

The number of cells to be seeded per well is dependent on the plating efficiency and proliferation rate of the cell line applied. For human lung epithelial A549 cells, with a rather high plating efficiency and doubling time of about 22 h, it is recommended to seed 30 cells/well in 0.5 ml of cell culture media for 12-well plates, or 50 cells in 1 ml media for 6-well plates. A sequential dilution of the cells to the right concentration is recommended. See the suggested procedure below:

a. Prepare dilution of 1 × 10^5^ cells/ml. Re-suspend well by pipetting and/or vortexing.
b. Prepare further 1 × 10^4^ cells/ml (10 × dilution of 1 × 10^5^ cells/ml) e.g. 0.1 ml of suspension of 1 × 10^5^ cells/ml plus 0.9 ml of medium. Vortex.
c. Prepare further 1 × 10^3^ cells/ml dilution e.g. 0.1 ml of suspension 1 × 10^4^ cells/ml plus 0.9 ml of medium. Vortex.
d. Prepare dilution of the number of cells you want per ml, e.g. 60 cells/ml (16.7 × dilution of l × 10^3^ cells/ml dilution).

Calculate the volume needed for all wells. It is recommended for more robust data to include six replicate exposure wells, three independent experiments. In case of shortage of test substances, the number of replicate wells can be reduced, but this will increase the margin of error. Place the cells in the incubator to settle before exposure to the test substance and controls. Remember to label the plate and the lid properly to avoid mix-up during the experiment.

### Preparation of test NM and controls

Prepare vials with 2x final concentrations of the test substance, diluted in cell culture media. Negative control is cells exposed to cell culture media only. A positive control should always be included to demonstrate responsiveness of the cells. This is especially important when non-cytotoxic results are obtained for the test substance. A good positive control would be e.g. chlorpromazine hydrochloride (50 µM) or staurosporine (200 nM). Concentrations to be applied should be tested for each cell line, as sensitivity will vary. A solvent control should also always be included. Test at least a concentration of the solvent used for the stock solution of the test substance equal to the solvent amount in the highest concentration of the test substance tested in the experiment. It is recommended to test also lower concentrations of the solvent and establish a concentration response curve.

Proper dispersion of the NM is required. Dispersion protocol needs to be optimized for each NM to be tested. The Nanogenotox protocol is a commonly applied dispersion protocol that works for many NMs and purposes (Jensen et al., 2011). As toxicity of NMs will depend upon physico-chemical properties, such as size, shape and surface coating, it is important always to perform physico-chemical characterization of the NM to be tested - both pristine material and in the actual dispersion.

### Exposure with NMs and controls

At 1–16 h after seeding of the cells, they are ready to be exposed. You should use about the same time after seeding for all your experiments for consistency. Negative control, solvent control, positive control and at least three concentrations of the test substance should be applied. It is preferred to include more than three concentrations of the NM tested for establishing a concentration response curve. It is recommended to include two sets of negative controls for increased robustness of the test method. For relatively non-cytotoxic compounds, it is important to test high enough concentration to be able to conclude about the effect. For standard chemicals, the maximum concentration for non-cytotoxic compounds should not be above 5 mg/ml, 5 ml/L, or 10 mM, whichever is the lowest. The concentration range should be selected regarding expected or demonstrated cytotoxicity, solubility in the test system, changes in pH or osmolarity. For NMs, up to 100 μg/cm^2^ should be used. This is equivalent to 380 μg/ml in 12-well plates (1 ml total volume) and 480 μg/ml in 6-well plates (2 ml total volume). However, for adherent cells, a dose metric per area is preferable. The highest concentration might be limited by agglomeration state of the NM to be tested.

Solvents and NMs suspension media with unknown effects should be also tested. A solvent control with the highest solvent concentration should be included in the assay. The stocks of test substances should then be prepared accordingly. The maximum solvent concentration depends on the type of solvent, but a general rule is that it should not exceed 5% for water, and 0.5% for solvents different from water or saline, (e.g. PBS and HBSS), such as methanol and DMSO.

A tip on how to choose concentrations: A linear range of concentrations (1, 2, 3, 4...) would normally be too tight, while a logarithmic range (1, 10, 100, 1000) is too much spread out. Steps of ∼3-fold (e.g. 1, 3, 10, 30, 100) are often just right.

Expose the cells by adding 0.5 ml (or 1 ml for 6-well) of cell culture medium with diluted test substance (2x final concentration) or control solution, so that in total you will have 1 ml medium/well for 12-well format or 2 ml medium/well for 6-well format. Leave the cells in the incubator for colonies to form. For A549 cells, 9–12 days is sufficient. Exposure could also be stopped after 72 h by removal of medium, washing 3x in PBS and adding new medium (2 ml for 6-well plates and 1 ml for 12-well plates), however it will not be possible to wash out all the NMs as they stick to the walls of the wells and to the cells (or are taken up). Thus, for NMs it is recommended to use continuous exposure for the length of the experiment, which is until colonies clearly visible by eyes are formed. For longer exposure time points than what is mentioned above, the cell culture media could be replaced with new medium, with or without the test substance, based on the experimental setting.

### Staining and counting of colonies

Colonies should be stained with 1% methylene blue. Add 20 µl methylene blue solution directly into the cell culture medium in each of the wells. Mix well by circular movements of the plate on the bench surface. Leave for minimum 30 min. The staining time can be increased if the staining is very weak. Pipette off all the medium with stain from all the wells. If needed, to reduce background staining, the plates can be rinsed carefully with water after staining but be careful not to wash off the colonies. Turn the plate upside down and leave on the bench to dry. Allow some air between the bench and the plate (e.g., place part of the lid under the edge of the plate).

Put the correct lid on each plate. Count the colonies from the bottom of the plate. Use an ink pen or a cell counter pen (e.g. e-count) to mark each counted colony to avoid double-counting. Only count colonies consisting of minimum 50 cells. Use a microscope to get familiar with selection of colonies sizes for counting. Create a template to note down the number of colonies for each well and each treatment group. Instead of manual counting, automatic counting equipment can be used (e.g. GelCount™ mammalian-cell colonies, spheroid and organoid counter, Oxford Optronix).

## Results

### Calculation of relative colony forming efficiency

**FIGURE 2:**
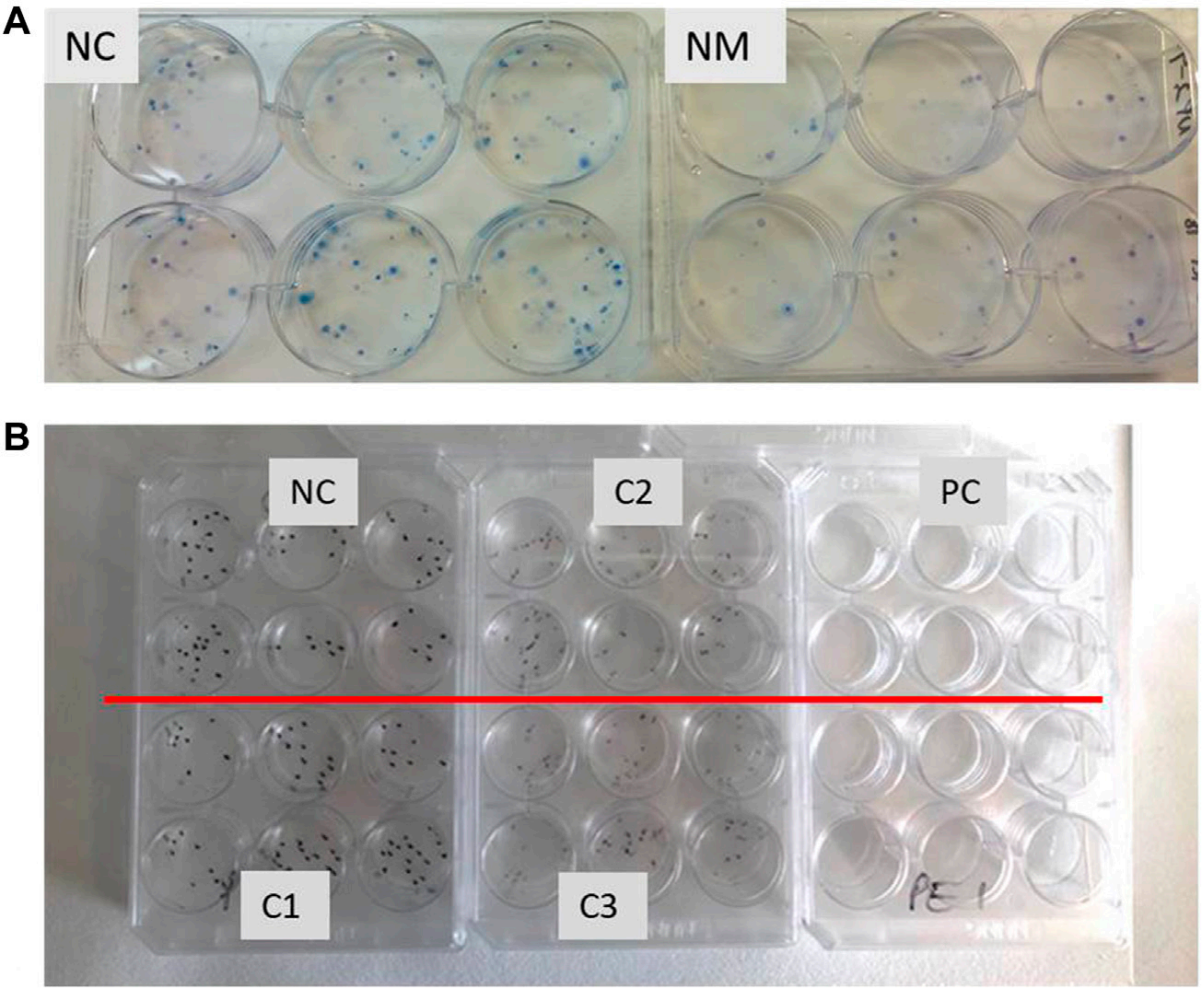
**(A)** Example of six well plates with cell colonies stained with methylene blue. A549 cells exposed to negative control (NC) and nanomaterial (NM), showing cytotoxic effect. Six replicate wells were exposed for each sample. **(B)** Example of 12 well plates with cell colonies stained with methylene blue. Each independent sample (negative control NC, positive control PC and tested compound with concentrations C1-low, C2-middle, C3-highest) has six parallels.

Each viable cell will form a colony (Figure 2). After counting the colonies, the CFE value is calculated as percentage based on the number of colonies formed relative to the number of inoculated cells, following the formula:

CFE (%) = (colonies counted/cells inoculated) x 100.

The relative CFE (RCFE) is the ratio of viability ratio between treated cells and negative control cells. Calculate RCFE as the number of colonies in the exposed sample normalized against the negative control, by using the mean of the replicates for each treatment group:

RCFE (%) = (average number of colonies in treatment plate/average number of colonies in negative control) x 100.

In addition to the number of colonies, a reduced colony size compared with control indicate a delay in the cell cycle. Thus, it is possible to distinguish between cytotoxic effects (reduction of the number of colonies formed) and cytostatic effects (reduction in colony size).

### Interpretation of results

When results are analyzed, it is important to compare with historical control data. Historical control data need to be logged for each laboratory, cell line and test method, and should include data for negative and positive controls to map baseline level for the cell line, as well as responsiveness.

Acceptance criteria for the experiments to be considered valid:

1. Exposure to the positive control must result in significant reduction (50%) or complete cell death (no colonies in the wells)
2. The plating efficiency in negative control should be comparable to historical control data for the specific cell line.

Criteria for characterizing the tested compound as cytotoxic are:

1. Cell viability (RCFE) is reduced by at least 20% compared to negative control
2. A concentration-dependent reduction in cell viability
3. Reproducible effects in at least three independent experiments

A test substance, for which the results do not meet the above criteria, is considered non-cytotoxic under the experimental conditions.

Statistical analysis could be used as an aid in evaluating the test results for example by a parametric or non-parametric statistical test for multiple comparison, such as ANOVA or Kruskal–Wallis test. This can be performed by a statistical software. To compare effects between various substances it can also be valuable to calculate effect concentrations, such as EC_50_-vaues or benchmark doses (e.g., EC_5_). This can be performed by non-linear regression analysis, such as the four parameter Hill-equation.

### Example of results

**FIGURE 3:**
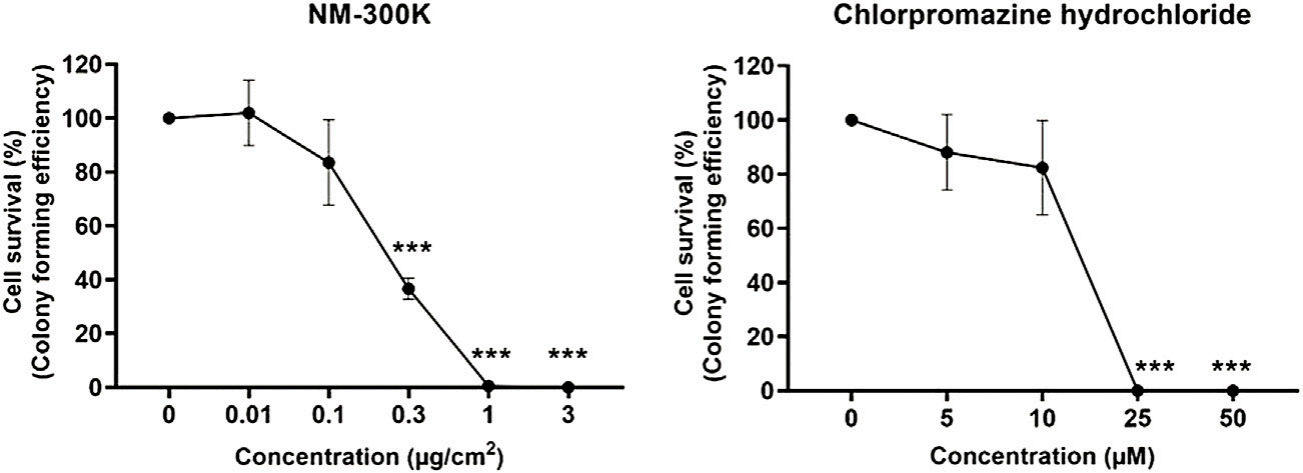
Relative colony forming efficiency (RCFE) on A549 cells exposed to nanosilver NM-300 K (JRC Nanomaterials Repository). Characterization information in El Yamani et al., 2017; Elje et al., 2020; Gábelová et al., 2017) (left image) and positive control chlorpromazine hydrochloride (right image) in 12-well plates. No colonies were seen at higher concentrations up to 75 μg/cm^2^ NM-300 K or 1,000 µM chlorpromazine hydrochloride (highest concentrations now shown). Results are presented as mean ± standard deviation of two independent experiments (n = 2), each with 12 replica wells for negative control (distributed in separate plates) and four replica wells for each concentration. Significantly different effects on cell survival compared to negative control (culture medium only) were analyzed by one-way ANOVA followed by Dunnett´s post-hoc test (***p < 0.001) in GraphPad Prism version 9.3.1 for Windows, GraphPad Software, San Diego, California United States.

In Figure 3, data from our laboratory are reported as an example of typical results that can be obtained by applying the 12-well plate CFE assay for testing of NMs and chemicals (respectively silver NM-300 K by the Joint Research Centre (JRC) Nanomaterials Repository and the positive control chlorpromazine hydrochloride).

## Discussion

The CFE assay is a very convenient test method for measuring cytotoxicity. It is shown to be highly compatible with testing of NMs, and free of interference since it is label-free. By miniaturizing it, the throughput is increased considerably. This makes the protocol suitable for cytotoxicity screening, although it takes some days for colonies to form. The assay is easy to perform, gives highly reproducible results, has low workload and low costs. Colonies can clearly be seen also without staining with methylene blue. However, one should note that nanoparticles can be present in the well, but those are easy to distinguish from colonies with more than about 50 cells, which is the size limit for counting. It is easy to recognize which colonies should be counted, and microscope will only be needed the first times to confirm that enough cells are present. Since the number of colonies formed is presented relative to the negative control, the most important thing is that the evaluation done with the manual counting is equal in all treatment groups, thus performed by the same person for all plates within an experiment. Ideally, the counting should be done “blinded” (with coded samples) to avoid performance bias. Quantification can also be performed by automatic colony recognition and counting. This will reduce potential bias introduced by subjective manual counting.

The test method protocol in this setting is not applicable for suspension cells, and not all adherent cells will form colonies with reasonable efficiency. Some cells are sensitive to cell density seeded and do not grow well if seeded too sparse. In our hands, the CFE assay works fine with the commonly applied cell lines A549, HepG2, JIMT-1, MDA-MB-231, T-47D and ARPE-19 (El Yamani et al., 2017; Buocikova et al., 2022, unpublished). Further, cells with exceedingly high doubling time will not be working so well with the CFE assay. For slowly growing cells and cells with low plating efficiency, the number of cells seeded should be increased, as well as the incubation time to form visible colonies. This should be tested and optimized for each cell line applied.

The seeding of cells is a critical phase for obtaining consistent results. The cells should be about 80% confluent before seeded for experiment, and the number of passages should be low (recommended below P15) to ensure high viability and avoid aging of the cell population. The cell suspension needs to be homogeneous, to ensure the same number of cells seeded in each well. It is also important to evenly spread the cells in the wells. If the variation between the wells is high, consider increasing the number of replicates. Normally, 4-6 replicate wells are sufficient.

Application of the CFE assay for toxicity testing of NMs was performed and validation was done by the JRC by interlaboratory comparison for the Petri dish format with 200 cells/dish (Ponti et al., 2009, 2014). The CFE protocol was in our laboratory firstly validated for six well plate format (El Yamani et al., 2017; Dusinska et al., 2019), and thereafter adapted to the 12 well format. Comparable results were seen when exposing 50 cells in six well plates as with 25–30 cells in 12-well format plates (not shown). The 6-well format protocol was recently successfully applied for NMs testing with A549 and HepG2 cells (El Yamani et al., 2017; Lee et al., 2022). It is beneficial to use the 12-plate format to increase the throughput and reduce the number of plates handled during an experiment. Use of 12-well plates will also reduce the amount of NMs needed, which can be critical when the supply of particles is short. The protocol has been thoroughly tested in our laboratory with a range of different NMs, including TiO_2_, SiO_2_, Ag, Au, BaSO_4_, CeO_2_, ZnO, CNT, graphene NMs (El Yamani et al., 2022), and liposomes, in A549 lung cells and also in liver HepG2 cells (data not shown).

NMs should be properly dispersed to avoid aggregation/agglomeration. Some materials can be difficult to disperse in the culture medium or may have a density that does not allow them to deposit on the surface. This is, however, a challenge that will apply for all *in vitro* models, which depend on exposures under submerged conditions. Cell-particle interaction should be assessed in any toxicological endpoint, especially when negative results are obtained.

Other cells found to be compatible with the assay are for example different human breast cancer cells and V79. The 12-well format protocol was standardized for application with NMs and validated by interlaboratory comparison by four laboratories in three different countries within the H2020 NMBP-13 RiskGONE project (paper in preparation). It is essential that the laboratory performing the test establishes historical control values for negative and positive controls for each cell line applied. Acceptance criteria for the test methods should be set based on historical control data, and plating efficiency and effects should generally be within mean ± 3 times the standard deviation, calculated from the historical control data for the cell line. Too large inaccuracy in cell seeding giving high variation in cell number seeded in the different wells, will introduce high variability in the RCFE values calculated. Since cell viability after treatment is calculated relative to negative control, it is of importance to have proper values for this. Thus, for more robustness, it is recommended to include two negative control plates in case one of them fails.

Exposure time can be continuous for the length of the experiment or stopped earlier. Ponti et al. (2014) reported exposure for 72 h and replaced the exposure medium with fresh culture medium. However, it is not possible to wash out all particles, as they normally stick to the plastic as well as to the cells, so continuous exposure is preferred.

It is recommended to calculate the effective concentration giving 50% reduction in cell viability (EC_50_ values) for better categorization of toxic potency of the test compounds.

It is important to report data in a harmonized and FAIR way (Jeliazkova et al., 2021). For several assays, including the CFE assay, data collection templates (with a function for automatic calculation of the results from the reported raw data) were developed within the RiskGONE project. The template is available upon request through the eNanomapper database, and it will be made publicly available. In this way, data from different laboratories can be compared and data can be used for meta-analyses.

The CFE assay is a sensitive assay for detection of cytotoxic effects, and as it is non-colorimetric and non-fluorescent it is especially applicable for testing of NMs to avoid interference between the NM tested and the readout or reagents of the assay, which is commonly seen with colorimetric or fluorometric assay e.g. the MTT, and other assays. Unlike most cytotoxicity assays which have an exposure time of less than 48 h, the CFE assay can be regarded as a sub-chronic assay since the exposure time is for several days, most often about 10 days. The CFE assay reflects true viability, i.e., the capacity of cells to proliferate. Thus, in the CFE assay, direct toxic effects on each cell are determined.

## Data Availability

The raw data supporting the conclusion of this article will be made available by the authors, without undue reservation.
